# Supplementary material for: Modeling the Copy Number of HSATII Repeats in Human Pericentromere
Source: Int J Mol Sci. 2025 May 15;26(10):4751. doi: 10.3390/ijms26104751 (PMC12112567; doi:10.3390/ijms26104751)
Supplement: Supplementary file 1 [file ijms-26-04751-s001.zip › ijms-3589400-supplementary.pdf]

Modeing the copy number of HSATII repeats in human pericentromere

Puranjan Ghimire[1], Richard I Joh[1,2*]

[1]Department of Physics, Virginia Commonwealth University, Richmond VA 23220

[2]Massey Cancer Center, Virginia Commonwealth University, Richmond VA 23220

*Corresponding author: Richard I Joh

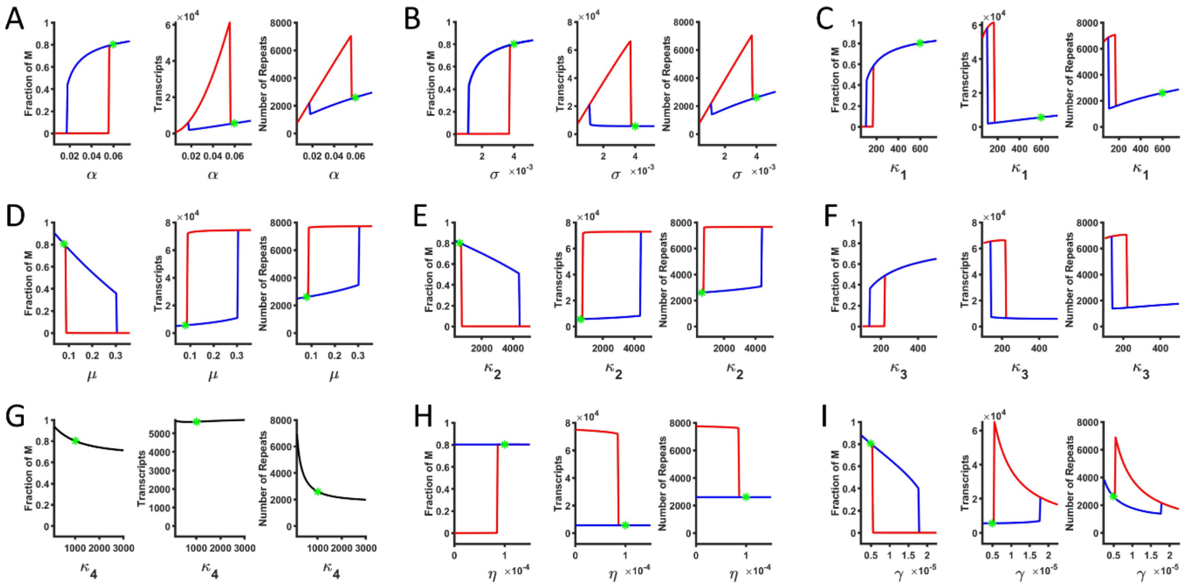

Figure S1: Steady-state solutions for fraction of methylated repeat, RNA, and copy number for the range of indicated parameters. The increase in parameters can promote copy gain (A-F) or copy loss (G-I). Blue and red curves indicate the desilenced and silenced state.

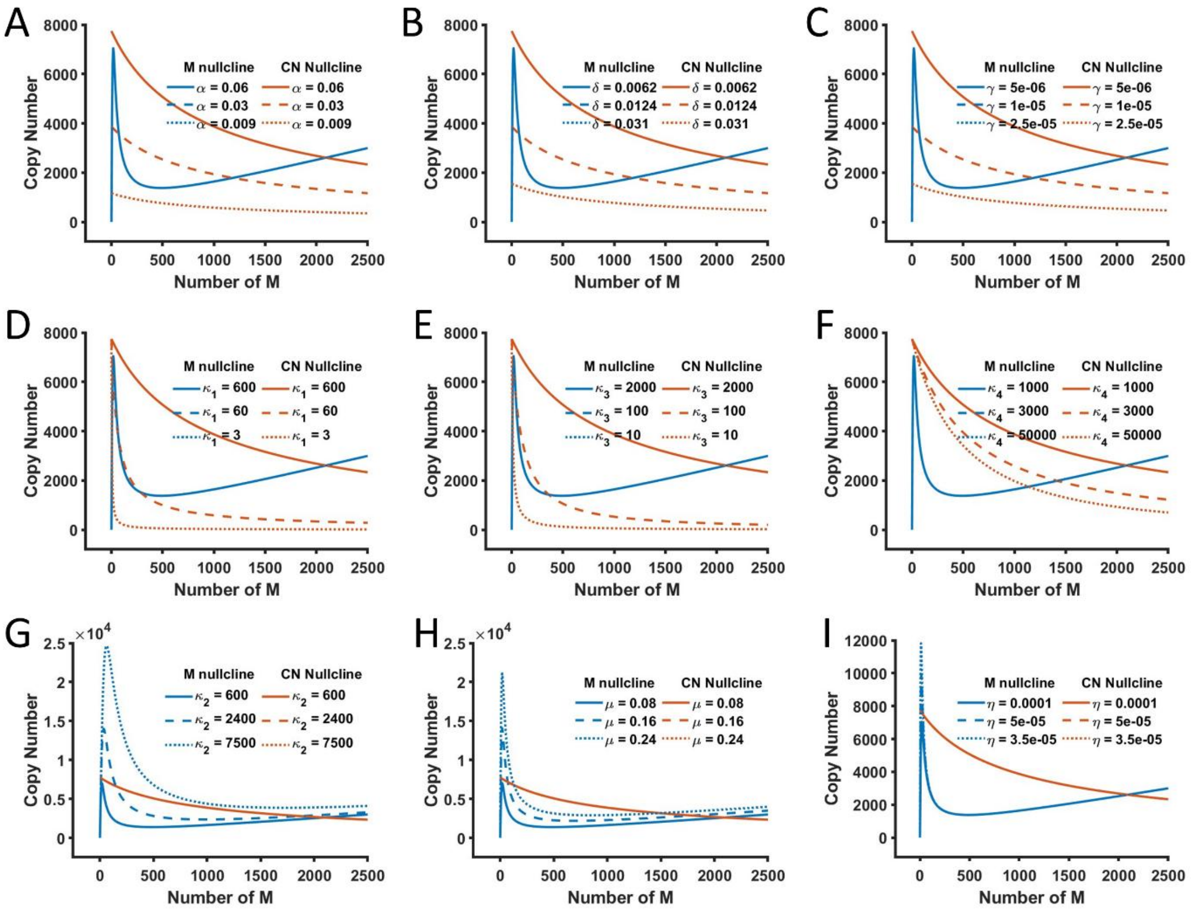

Figure S2: Nullcline analysis for QSSA when varying one parameter. (A-F) Change in parameters affects the CN Nullcline. (G-I) Change in parameters affects the M nullcline. In all the figures, the solid line represents the nullcline at reference value.

Table S1: Parameter values used for solving Eqs. 1-3.

Parameters	Values for WT cells	References
Transcription rate (α)	$0.06\ min^{-1}$	*Paolo Maiuri et. al., EMBO Reports, 2011*
Half maximum saturation number of M for transcription (κ_1)	600	
RNA degradation rate (δ)	$0.0062\ min^{-1}$	*Kaiwen Shi, et. al. Plos Comp. Biol. 2021*
Cooperative conversion rate of U to M (ϕ)	$0.0002\ min^{-1}$	
Half maximum saturation number of M for cooperative methylation (κ_2)	600	
Spontaneous conversion of U to M (η)	$0.0001\ min^{-1}$	

Spontaneous conversion rate of M to U (μ)	$0.08\ min^{-1}$	
Reverse transcription rate (σ)	$0.004\ min^{-1}$	
Half maximum saturation number of M for reverse transcription (κ_3)	2000	
Repeat recombination rate (γ)	$0.000005\ min^{-1}$	
Half maximum saturation number of M for reverse transcription (κ_4)	1000	

Table S2: Initial conditions applied in the simulation (Equations 1-3)

**S. N.**	**Initial Conditions (RNA, M number, Copy Number).**
1	(0, 10, 20)
2	(2.04e4, 779, 5212)
3	(10000, 1000, 6000)
4	(20000, 500, 5000)
5	(2000, 1000, 5000)
6	(500, 500, 10000)
7	(0, 2000, 10000)
